# Differential Expression Profile of Salivary oncomiRNAs among Smokeless Tobacco Users

**DOI:** 10.1055/s-0043-1761191

**Published:** 2023-02-22

**Authors:** Natheer H. AL-Rawi, Zuha Rizvi, Sarra Mkadmi, Rawan Abu Kou, Neibal Elmabrouk, Mohammad S. Alrashdan, Aghila Rani Koippallil Gopalakrishnan

**Affiliations:** 1 Department of Oral & Craniofacial Health Sciences, College of Dental Medicine, University of Sharjah, Sharjah, United Arab Emirates; 2 Sharjah Institute of Medical Research, University of Sharjah, Sharjah, United Arab Emirates

**Keywords:** smokeless tobacco, miR-21, miR-146a, miR-155, miR-199a, oral cancer

## Abstract

**Objective** The aim of this study was to evaluate the expression of selected salivary oncomiRNAs among smokeless tobacco users and nonsmokers.

**Materials and Methods** Twenty-five subjects with chronic smokeless tobacco habit (> 1 year) and 25 nonsmokers were selected for this study. MicroRNA was extracted from saliva samples using the miRNeasy Kit (Qiagen, Hilden, Germany). The forward primers used in the reactions include hsa-miR-21-5p, hsa-miR-146a-3p, hsa-miR-155-3p, and hsa-miR-199a-3p. Relative expression of miRNAs was calculated using the 2-ΔΔCt method. Fold change is calculated by raising 2 to the power of the negative ΔΔCT value.

**Statistical Analysis** Statistical analysis was carried out using GraphPad Prism 5 software. A *p* -value less than 0.05 was considered statistically significant.

**Results** The four tested miRNAs were found overexpressed in saliva of subjects with smokeless tobacco habit when compared with saliva from nontobacco users. miR-21 expression was 3.74 ± 2.26 folds higher among subjects with smokeless tobacco habit compared to nontobacco users ( *p* < 0.01). The expression for miR-146a (5.56 ± 8.3 folds; *p* < 0.05), miR-155 (8.06 ± 23.4 folds; *p* < 0.0001) and miR-199a (14.39 ± 30.3 folds; *p* < 0.05) was significantly higher among subjects with smokeless tobacco habit.

**Conclusion** Smokeless tobacco leads to salivary overexpression of the miRs 21, 146a, 155, and 199a. Monitoring the levels of these four oncomiRs may provide insight about the future development of oral squamous cell carcinoma, especially in patients with smokeless tobacco habits.

## Introduction

Oral cancer is the fourth leading cause of death in the Middle East and North Africa (MENA) area, where it is a major contributor to the overall burden of disease. New instances of oral cancer are reported every year across the world. 1 2 Alqahtani et al reported that the Gulf Cooperation Council (GCC) countries, Saudi Arabia leading, followed by the United Arab Emirates (UAE), had the highest incidence and fatality rates for oral and oropharyngeal malignancies. 3 The UAE was second in terms of age-standardized incidence rates and oral cancer-associated age-standardized death rates. 3

Out of the many possible causes of oral cancers, 90% can be attributed to preventable ones, some of which are alcohol consumption, smoking, and the use of smokeless tobacco (SLT). In the GCC, the overall prevalence of oral and oropharyngeal cancers due to the use of SLT products is 91.9%. 3 SLT is widely used in the UAE, Naswar and pan chewing being the most common forms. 4 Some of the other types include chewing tobacco, snuff, gutkha, betel quid with tobacco, and toombak. Naswar is synthesized as a mixture of dried tobacco leaves, calcium hydroxide paste, wood ash and other flavorings. The occurrence of cancers of various sites, including oral, has been studied globally and a significant correlation is reported between the use of SLT and the risk of oral cancer. 5 For a long time, SLT products have been under research scrutiny for their effect on the risk of developing oral cancers. 6 Over 30 carcinogens have been recognized in SLT products including tobacco-specific nitrosamines that are known to be potent carcinogens. SLT products also display signs of dependence like in cigarette smokers but pose a markedly different threat that may be due to the diversity in the product types and the variety in their composition. 4 The use of Naswar has been associated with cancers of the oral cavity as well as esophageal cancers. 5

Micro-RNAs (miRNAs) play an essential role in the modulation of gene expression and several studies are ongoing to explore their efficacy as potential biomarkers for health and diseases. 7 Recent studies highlight the potential use of miRNAs as promising noninvasive biomarkers for a variety of malignancies, including breast, oral, and lung cancers. 8 Many research have established the efficacy of miRNAs as key regulators of several cellular processes including embryonic development, cell proliferation, cell growth, tissue differentiation, and apoptosis. 9 Additionally, miRNAs participate in the cellular signaling network, differential gene expression, and the coregulation of transcription factors. 10

The gold standard for the confirmation of oral squamous cell carcinoma (OSCC) remains the conventional oral examination, but salivary biomarkers have been making an increasing appearance. 11 Saliva proves to be an excellent medium for the extraction of miRNAs considering that it is noninvasive, inexpensive and easy to collect and store which makes it ideal for screening large populations. 12 13 Saliva can be used as a diagnostic tool with highly sensitive and specific markers. According to meta-analysis study done by Hema Shree et al, certain salivary biomarkers were measured and determined to be highly sensitive and specific for detecting OSCC, with some reaching 100% in accuracy. These include the proteins MMP-9 and chemerin as well as the miRNA biomarkers 274 and 136. 14 Considering that the International Agency for Research on Cancer has declared that adequate evidence exists proving the carcinogenicity of SLT, utilizing salivary miRNA for research into health and disease in SLT users is an endeavor of interest. 15 The importance of salivary miRNA in the diagnosis and prognosis of OSCC and cases of periodontitis has recently been discussed in our recent publications. 16 17 Oral malignancies of various grades and stages were shown to have altered levels of salivary miRNA, where they were either up- or downregulated. 16 miR-21, miR-146a, miR-155, and miR-199a are found to play an important role in head and neck squamous cell carcinoma (HNSCC). 18 19 20 21 The overexpression of miR-155 promotes the proliferation, migration, and invasiveness of OSCC cells through controlling the BCL6/cyclin D2 axis. 18 miR-21, on the other hand, promotes carcinogenesis via its antiapoptotic effect. 22 miR-146a targets IRAK1 and TRAF6 to promote nuclear factor-κB (NF-κB) signaling in oral, cervical, breast, and prostate cancers. 21 22 23 24 25 Overexpression of miR-199a-5p suppressed invasion and migration of OSCC cells through blocking the SOX4/EMT pathway. 26 The objective of this study was to examine the expression of targeted salivary miRNAs between SLT users and nonusers and the potential of these salivary miRNA biomarkers as early diagnostic indicators for HNSCC among SLT users.

## Materials and Methods

### Identification of Candidate miRNA Genes Associated with Head and Neck Squamous Cancer

To identify candidate miRNAs that can serve as potential salivary biomarkers of HNSCCs, we performed a comprehensive search using publicly available databases. Evidence for association of human miRNAs with various cancers was searched and downloaded from dbDEMC website ( https://www.biosino.org/dbDEMC/index ). dbDEMC database is a publicly available database of differentially expressed miRNAs in human cancers. 27 We were able to identify expression levels of miR-21 (source: TCGA), miR-155 (source: TCGA), miR-146 (source: TCGA), and miR-199 (source: GEO) in association with head and neck squamous cancers and their expression levels are presented in supplementary material Table S1 .

### Sample Collection

The saliva samples were collected at a single point of time using a cross-sectional quantitative study design. The design and nature of the study were explained to the participants and informed written consent for their participation was procured. The study procedure was approved by the University of Sharjah's Research Ethics Committee (REC-19-09-08-01-S) and all methods and procedures were performed in accordance with the relevant guidelines and regulations. Volunteers using smokeless tobacco (N = 25) and nonusers of smoke/smokeless tobacco (N = 25) were selected from male UAE residents of Southeast Asian descent over the age of 15 who visited the University Dental Hospital, University of Sharjah. The saliva samples were collected and transferred in an ice container, aliquoted, and kept at −80°C until further analysis.

### Extraction of Salivary miRNA

Following the manufacturer's instructions, miRNA was extracted from entire saliva samples (about 300 μL) using the miRNeasy Serum/Plasma Kit (QIagen, Germany). Nanodrop was utilized to evaluate the quality and amount of miRNA samples (ND2000; Wilmington, DE, United States). Complementary DNA was synthesized using MystiCq® miRNA cDNA Synthesis Mix (MIRRT-100RXN, Sigma, United States). Briefly, 10 μL poly (A) tailing reaction mixture contained 1 μg of miRNA, 2 μL of 5x poly A tailing buffer, 1 μL of Poly (A) polymerase and molecular grade water to adjust final reaction volume. The polyadenylation reaction was carried out at 37°C for 60 minutes followed by 70°C for 5 minutes. First-strand cDNA synthesis reaction was then set up using 10 μL of poly (A) tailing reaction mix, 9 μL of mystiqCq miRNA cDNA reaction mix, and 1 μL of Readyscript reverse transcriptase. The reverse transcriptase reaction was initially performed at 42°C for 20 minutes followed by 85°C at 5 minutes. The cDNA was then subjected to real-time polymerase chain reaction (PCR) analysis for quantification of expression of miR-21, 146a, 155, and 199a (Sigma, United States) in the study samples. The forward primers used in the reactions include hsa-miR-21-5p, hsa-miR-146a-3p, hsa-miR-155-3p, and hsa-miR-199a-3p. MystiCq miRNA primer RNU6A (Sigma, United States) was used as the universal reverse primer in all the reactions. Small nuclear RNA (snRNA) U6 (Sigma, United States) was used as the housekeeping gene for data normalization. Real-time PCR was carried out in StepOne real-time PCR thermocycler (Applied Biosystems, United States) in a total reaction volume of 25 μL using MystiCq SYBR Green ready mix with carboxy-X-rhodamine (ROX) (Sigma, United States). Each sample was tested in duplicates.

### Real-Time PCR Analysis

Relative expression of miRNAs was calculated using the 2-ΔΔCt method. Reactions were performed using specific forward and universal reverse primers along with MystiCq SYBR Green ready mix based on the manufacturer's protocol. The cycle threshold value, Δct, was calculated for each sample and normalized using housekeeping snRNA U6. The ct values used for analysis were less than 35 and a nontemplate control was used as a negative control. The mean ct values of each target miRNA and reference gene U6 in the control (nonsmoker) group were used in calculating the ΔΔCT value. Fold change was calculated by raising 2 to the power of the negative ΔΔCT value.

### Statistical Analysis

Statistical analysis was carried out using GraphPad Prism 5 software. Data was presented as mean ± standard deviation. Statistical differences between the study groups comparing the expression of the miRNAs were calculated by Mann–Whitney U test. A *p* -value less than 0.05 was considered statistically significant.

## Results

This study consisted of 50 subjects (25 nontobacco users and 25 SLT users). All were on Naswar habit for more than 1 year. Differential expression of miR-21, miR-146a, miR-155, and miR-199a was studied and compared among the study groups by real-time PCR analysis. MicroRNA expression was quantified by delta ct values, where Ct = threshold cycle, delta Ct = (Ct target miRNA minus Ct RNU6) and the relative expression of miRNA was calculated using the 2-ΔΔCt method. By means of the ΔΔCt equation, the expression of experimental miRNAs in the saliva samples of SLT users was calculated in comparison to the nonusers using the endogenous control RNU6B. Mean delta ct values of the four miRNAs expressed among the study participants are given in Table 1 .

**Table 1:** Mean delta ct values (mean ± SD) of miR-21, miR-146a, miR-155, and miR-199a in the saliva samples of SLT users

	miR-21	miR-146a	miR-155	miR-199a
**SLT users**	0.82 ± 2.32	-12.83 ± 2.64	9.29 ± 2.31	1.88 ± 3.77
**Nontobacco users**	−8.74 ± −9.29	−12.13 ± 2.14	0.20 ± 2.28	2.64 ± 2.13
***p*** **-Value**	0.028	0.29	*p* < 0.0001	0.9847

Abbreviations: ct, cycle threshold; SD, standard deviation; SLT, smokeless tobacco.

### miR-155 Is Differentially Expressed in Smokeless Tobacco Users Compared to Nonsmokers

**Fig 1:**
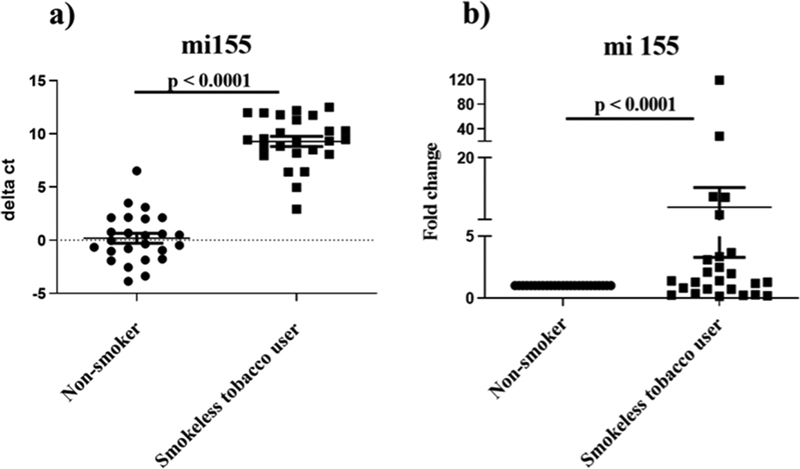
Expression of miR-155 in saliva samples of smokeless tobacco (SLT) users in comparison to nonusers of smoke/smokeless tobacco. ( **A** ) Plot representing the delta cycle threshold (Ct) comparing the miR-155 expression in SLT users compared to nontobacco using controls. Horizontal bars represent the mean and range of the delta Ct for all the samples. ( **B** ) Fold change in expression of miR155 among SLT users compared to nontobacco using controls.

Among the four miRNAs studied in this study, expression of miR-155 was found to be significantly upregulated among all the users of SLT enrolled in the current study compared to nontobacco using controls. The ct values obtained for miR-155 from each subject were standardized using the housekeeping gene (U6) that is used as the normalization control. When the mean delta ct values of the study samples were compared, a significant difference was observed in the smokeless tobacco users compared to nonusers (9.29 ± 2.31 vs 0.20 ± 2.28; *p* < 0.0001; Fig. 1A ). Similarly, we found that miR-155 expression was eightfolds higher in SLT users compared to nonusers (8.06 ± 23.4; *p* < 0.0001; Fig. 1B ). The data suggests the inflammatory status of the oral cavity of SLT users.

### Differential Expression of miR-21, miR-146a, and miR-199a among Users of SLT

**Fig 2:**
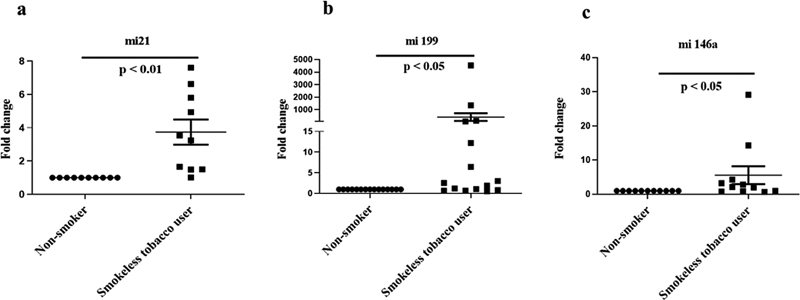
Expression of miR- 21, miR-199, and miR-146a in saliva samples of smokeless tobacco (SLT) users in comparison to nonusers of smoke/smokeless tobacco. Fold change in expression of ( **A** ) miR-21, ( **B** ) miR-199, and ( **C** ) miR-146a in the saliva samples of SLT users compared to nonusing controls.

Among the 25 saliva samples from SLT users analyzed in this study, 10 (40%), 16 (64%) and 11 (44%) samples demonstrated significantly enhanced expression of miR-21, miR-199, and miR-146, respectively. The expression for miR-21 was found to be 3.74 ± 2.26 folds higher ( *p* < 0.01; Fig. 2A ) among SLT users compared to the nontobacco users. Similarly, miR-199 was enhanced by14.39 ± 30.3 folds ( *p* < 0.05; Fig. 2B ) and miR-146 was upregulated by 5.56 ± 8.3 folds ( *p* < 0.05; Fig. 2C ).

## Discussion

The incidence of HNSCC has increased due to the scarcity of early diagnostic tools. Oral cancer and its early detection are the subject of intense research since it is the fourth greatest cause of mortality in the MENA region. In addition to other benefits, the fact that salivary miRNA is noninvasive and affordable makes it a promising tool for early detection. Given that miRNA expression has been shown to be potentially useful as a biomarker in early oral cancer diagnosis, 16 the purpose of this study was to examine the expression of preselected miRNAs in SLT users versus healthy controls. The study investigated the expression of the four salivary miRNAs such as miR-21, 146a, 155, and 199a.

According to Lubov et al, several miRNAs have a significant role in the regulation and expression of genes in HNSCC, either as oncogenes or tumor suppressors. 28 miR-21 is one of the first oncomiRs discovered to be increased in a number of malignancies, including gliomas, breast cancer, and colorectal cancer. 29 miR-21 is overexpressed in HNSCC and targets the actin-binding protein tropomyosin 1, which is essential for maintaining the cell skeleton. Deregulation of this miRNA will promote malignant transformation that evade immune recognition. 30 Overexpression of the oncogene miR-21 results in malignant B-cell lymphomas. 31 miR-146a inhibits both the interleukin-1 receptor associated kinase 1 (which is implicated in the NF-κB pathway) and the epidermal growth factor receptor. These receptors contribute to the development of various cancer in humans. Therefore, in HNSCC, expression of this miRNA is decreased. 32 33 34 35 However, miR-146a-5p is expressed in the serum and tissue samples of small cell lung cancer patients. 36 miR-155 is a key regulator of cyclin D2 and BCL6. Cyclin D2 is a protein that stimulates cell division, whereas BCL6 is a transcriptor that inhibits cell division. Consequently, miR-155 is shown to be overexpressed in malignancy, boosting Cyclin D2 and lowering BCL6 expression directly. 37 38 Upregulated levels of expression of miR-155 along with miR-21 was observed in premalignant lesions and invasive pancreatic cancers compared to the normal tissues. 19 miR-199a-5p, on the other hand, is downregulated in human OSCC tissues and cell lines, and its expression is lower in OSCC tissues with lymph node metastasis compared to tissues without lymph node metastasis. In addition, the findings of Wei et al demonstrate that miRNAs suppress OSCC cell invasion and migration by targeting the oncogene SOX4. 26 The reason for selecting and evaluating these four miRNAs is their participation in the aforementioned critical and different pathways. This supports the hypothesis that SLT users have a high level of inflammation in their oral cavity, given that miR-155 is strongly associated with inflammatory response and its expression is elevated in cancer cells. 39 In addition, Hu et al demonstrated that miR-155 overexpression regulates many cancer-related pathways involving cell proliferation, invasion, migration, stemness, and angiogenesis. 40 This agrees with the current findings that miR-155 was overexpressed in all the SLT users enrolled in the study in comparison with the nonsmokers. Likewise, in agreement with the literature, miR-21 was found to be overexpressed in 40% of SLT users in this study. miR-21regulates the development, differentiation, and death of normal cells in addition to influencing carcinogenesis. 31 Similarly, in this study, we report that 44 and 64% of SLT users had elevated levels of miR-146 and miR-199a in the oral cavity compared to nonusers, in contrast to earlier results indicating their downregulated levels in HNSCC patients. 26 32 33 34 35

The frequency of use of SLT and the expression of the investigated miRNAs were not correlated in this investigation. Additionally, the presence of oral lesions had no discernible influence on the outcomes. Overall, the results of the study suggest that miR-155 could be considered as an effective diagnostic biomarker indicating predisposition of oral cancers among SLT users. To assess the diagnostic use of miR-21, miR-146, and miR-199, more research is nonetheless required. Participants were recruited from a single center that constitutes one of the major limitations of this study. There was also no long-term monitoring or follow-up of SLT employing individuals with lesions to observe or report the development of malignancies or the maintenance of oral health. Further long-term follow-up studies are needed to check the expression pattern of these miRNAs especially among patients developing SLT-related oral disorders.

## Conclusion

SLT leads to salivary overexpression of the miR-21, miR-146a, miR-155, and miR-199. Monitoring the levels of these oncomiRs may provide insight about the future development of OSCC especially in patients with SLT-related potentially malignant lesions.
