# Supplementary material for: Differential Expression Profile of Salivary oncomiRNAs among Smokeless Tobacco Users
Source: Eur J Dent. 2023 Feb 22;17(4):1215–20. doi: 10.1055/s-0043-1761191 (PMC10756836; doi:10.1055/s-0043-1761191)
Supplement: Supplementary file 1 — Supplementary Material [file 10-1055-s-0043-1761191-s2282331.pdf]

Supplementary Material Table S1 Differential expression levels of miR-21, miR-155, miR-146, and miR-199 in head and neck cancers. Source: https://www.biosino.org/dbDEMC/index

Cancer type	Design	Sample case	miRID	logFC	AveExpr	t	*p*-Value	B	No of samples	Source
Head and neck cancer	Cancer vs. normal	HNSC	hsa-miR-21-5p	1.567439198	17.95079	17.57629	1.58E-55	115.5597	564	TCGA
Head and neck cancer	Cancer vs. normal	HNSC	hsa-miR-155-3p	0.503668701	−0.775638	3.513393	0.000478	−1.385718	564	TCGA
Head and neck cancer	High-grade vs. low-grade	HNSC stage 3	hsa-miR-146a-3p	3.023390464	−1.486151	9.824656	3.90E-21	37.23433	564	TCGA
Head and neck cancer	Poor outcome vs. good outcome	Short time survival	hsa-miR-199a-3p	−2.69658317	9.03946	−6.889757	3.09E-08	8.826223	40	GEO

Abbreviation: SC, squamous cancer.

 © 2023. The Author(s).
